# Ectopic Pituitary Neuroendocrine Tumor: A Case Report Written With the Help of ChatGPT

**DOI:** 10.7759/cureus.46999

**Published:** 2023-10-13

**Authors:** Jun Li, Jiansheng Zhong, Ziqi Li, Yong Xiao, Shousen Wang

**Affiliations:** 1 Neurosurgery, Department of Neurosurgery, Fuzhou 900th Hospital, Fuzong Clinical Medical College of Fujian Medical University, Fuzhou, CHN; 2 Neurosurgery, Department of Neurosurgery, Oriental Hospital Affiliated to Xiamen University, Fuzhou, CHN; 3 Neurosurgery, Central Institute for Mental Health, University of Heidelberg, Heidelberg, DEU

**Keywords:** ectopic pituitary neuroendocrine tumor, artificial intelligence, neuro-oncology, brain tumor, chatgpt

## Abstract

ChatGPT (Chat Generative Pre-trained Transformer) is a chat robot program developed by OpenAI (OpenAI Incorporated, Mission District, San Francisco, USA). It is a natural-language processing tool driven by artificial intelligence technology. It can complete tasks such as writing emails, video scripts, copywriting, translation, coding, and writing papers. At present, several studies have been conducted on the use of this tool for academic writing, so we report a rare case of suprasellar ectopic pituitary neuroendocrine tumor (PitNET) secreting growth hormones, in a female 53-year-old patient with facial changes and hypertrophy of her hands and feet for six years. When asking questions on unusual topics, ChatGPT can provide answers but not specific ones, and similar to most machine learning solutions, verifying the correctness of information is a problem.

Question asked from ChatGPT: Summarize ectopic pituitary neuroendocrine tumor.

“Suprasellar heterotopic pituitary neuroendocrine tumors refer to tumors formed by the heterotopic growth of pituitary neuroendocrine tumor cells in the sellar region other than the pituitary glands. This tumor is common in middle-aged women, and it often manifests as headache, visual impairment, and endocrine dysfunction. Diagnosis is usually made by combining history, physical examination, blood tests, and imaging. Treatment is primarily performed through surgical resection of the tumor, radiotherapy, and drug therapy, and the prognosis is mostly good.”

The content in quotation marks is generated by ChatGPT.

## Introduction

ChatGPT (Chat Generative Pre-trained Transformer; OpenAI Incorporated, Mission District, San Francisco, USA) is a state-of-the-art large language model (LLM) based on the GPT-4 architecture [1]. Since its initial public release in November 2022, subsequent versions, such as GPT-4, have increasingly been equipped with artificial intelligence-guided dialog (AIGC) functionality and have gradually confirmed that they may have the potential to assist with medical education and, potentially, clinical decision-making [2]. Ectopic pituitary neuroendocrine tumor (PitNET) is a rare disease that refers to a pituitary neuroendocrine tumor located outside the sella turcica without any connection with the intrasellar components, and the pathogenesis remains unclear [3]. The clinical manifestations of ectopic PitNET depend on the type and level of secreted hormones and the compression of adjacent structures by the tumor [4]. The diagnosis of ectopic PitNET is primarily based on clinical manifestations, endocrine examinations, and imaging findings [5]. For patients with masses along the transitional distribution of Rathke’s cysts with normal intrasellar images and evident endocrine changes of functional pituitary tumors, the possibility of ectopic pituitary tumors should be considered [5]. Considering that ectopic PitNETs are rare, information about their epidemiology, clinical presentation, and management is limited. In this report, we aim to provide the existing knowledge of ectopic PitNET, including epidemiology, clinical presentation, diagnosis, management, and prognosis, using the natural language processing-based machine learning model ChatGTP.

## Case presentation

**Figure 1:**
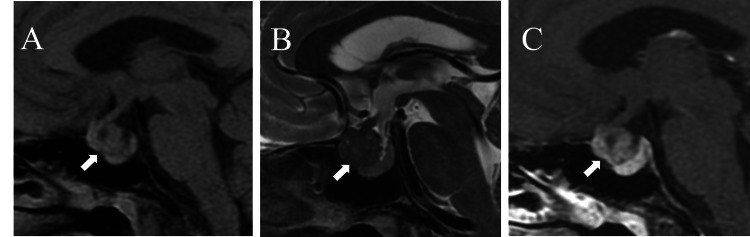
Preoperative MRI of the pituitary gland (A) Sagittal position on T1WI, a round-like isointense shadow could be observed in the suprasellar region; (B) sagittal position on T2WI, a round-like low-signal shadow can be seen; (C) sagittal position of T1 contrast enhancement. A round-like soft tissue mass was observed in the suprasellar region with mild enhancement (white arrow), clear boundary, and pituitary gland located at the posterior and inferior parts of the lesion

**Figure 2:**
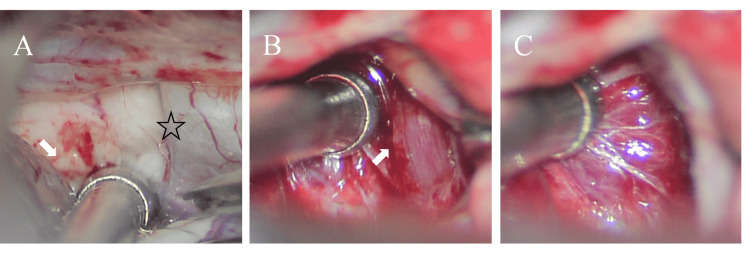
Microscopic findings during operation using the supraorbital lateral approach (A) Tumor in the anterior cruciate space (white arrow), right optic nerve (asterisk); (B) complete pituitary stalk (white arrow); (C) the sellar diaphragm is intact, and no evident manifestation of tumor origin is observed

**Figure 3:**
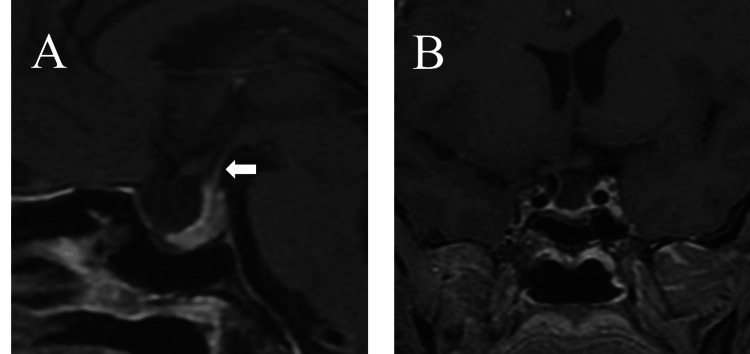
MRI of the pituitary gland reexamined seven days after surgery (A) Sagittal position on T1WI, complete pituitary stalk (white arrow); (B) coronal position on T1WI, complete resection of the tumor was observed

**Figure 4:**
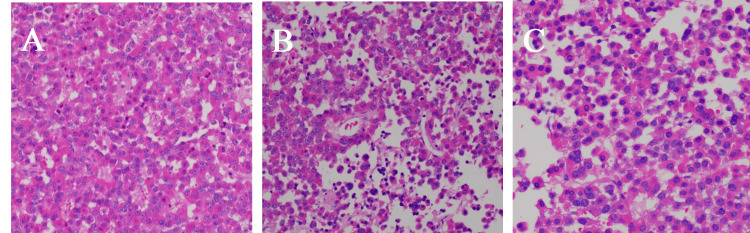
Postoperative histopathological sections (A) Tumor cells can present a glandular tubular structure; (B) visible perivascular pseudorosette; (C) acidophilic fine particles can be seen in the cytoplasm

A 53-year-old woman presented with facial changes and hypertrophy of her hands and feet for six years. Physical examination upon admission showed hypertrophic nasal lip, widened nasal ala, prominent forehead and mandible, prominent zygoma, and hypertrophic extremities were observed. No evident abnormality was found in the rest of the physical examinations. After admission, hormone examination showed that the fasting random growth hormone level was 10.5 ng/ml, insulin-like growth factor 1 (IGF-1) was 484 ng/ml, free thyroxine (FT) 49.64 pmol/L, and the trough value of growth hormone was 10.5 ng/ml after oral glucose administration. Other hormone levels were normal. MRI examination of the pituitary gland revealed a quasi-circular, space-occupying lesion in the suprasellar region, with a slightly hypointense signal on T2WI and a slightly hyperintense signal on T1WI. After enhancement, the lesion showed mild to moderate uniform enhancement, the boundary with the pituitary and pituitary stalk was clear, and the optic chiasm was pressed superiorly (Figures 1A-1C). Four days after admission, a supraorbital lateral approach to pituitary neuroendocrine tumor resection was performed. During the operation, the tumor was white, with poor blood supply and soft texture (Figures 2A-2C). Except for surgical resection of the tumor, no radiation or hormone therapy was performed. Her visual acuity and visual field did not change postoperatively. A reexamination of MRI seven days postoperatively revealed complete tumor resection (Figures 3A, 3B). Postoperative pathology showed diffuse growth of the tumor, some areas were sinus-like, with a glandular-like arrangement (Figure 4A) and perivascular pseudorosette formation (Figure 4B). The tumor cells were consistent, the heteromorphic type was not obvious, the size was medium, the shape was round or oval, the cytoplasm was rich, most of the cytoplasm was eosinophilic, a small part of the cytoplasm was chromophobe or slightly eosinophilic, and the nucleolus and mitotic figures were rare (Figure 4C). Immunohistochemistry showed that it was a multi-hormone cell adenoma, expressing transcription factors PIT-1 and SF-1. Postoperative random values for growth hormone and insulin-like growth factor decreased to 0.365 and 420 ng/ml, respectively, and the remaining hormones were normal. After two years of follow-up after surgery, the patient did not show any neurological dysfunction.

## Discussion

An ectopic pituitary neuroendocrine tumor(PitNET) originates from the residual embryo tissue of pituitary tissues in the pharyngeal region and the residual tissue of Rathke’s capsule during embryonic development and migration (from the nasopharynx to the sella turcica) [6]. The incidence rate is only 0.7%-2.4% of that of pituitary neuroendocrine tumors [7]. Ectopic PitNET can occur intracranially and extracranially, with approximately 73% of tumors located in the sphenoid sinus or suprasellar region while other areas include the clivus, cavernous sinus, paraumbrella, nasal cavity, nasopharynx, and third ventricle [8]. Ectopic PitNET on the saddle often needs to be differentiated from meningioma of the sellar tuberosity [9]. Although there are many similarities between the two, there are still some important differences in clinical manifestations, age, and prognosis [10,11]. There is no significant gender difference in patients with ectopic PitNET, and symptoms depend on the involvement of adjacent structures and hormone activity [10]. Nodular sellar meningioma is more common in women, often with asymmetric visual impairment [11]. In addition, ectopic PitNET that occurs in different regions needs to be distinguished from other lesions in the corresponding region, such as in the clivus, which needs to be distinguished from chordoma, meningioma, plasma cell tumor, etc [12]. The endoscopic PitNET of the sphenoid sinus and nasal cavity needs to be differentiated from metastatic tumors, other neuroendocrine tumors, nasopharyngeal carcinoma, etc. [12].

Characteristic imaging features of ectopic PitNET

On CT, it is often as dense as gray matter and moderately enhanced imaging, which can be used to evaluate bone erosion [13]. MRI shows that the tumor is a mass located in an area other than the pituitary gland, and it is not connected to the normal pituitary gland in the sellar region [13]. Based on the growth space, the tumor may be round or irregular in shape with a slightly lower or isointense signal on T1WI and an isointense or slightly high signal on T2WI, the internal signal is mostly uneven [5]. The enhanced scan may show no evident enhancement or uneven enhancement, and the degree of lesion enhancement is lower than that of the normal pituitary gland [5]. Some lesions may present uneven punctiform long T1 and long T2 signals, which are enlarged lacunae in the tumor with secretion accumulation therein, and the solid parts without enhancement and with evident enhancement of the tumor form a “sieve pore-like” structure [13]. Some ectopic PitNET may undergo secondary changes such as hemorrhage, necrosis, and cystic degeneration, or may be accompanied by the vacuolation of the sella turcica [14].

Similar to typical pituitary neuroendocrine tumors, surgery is the treatment of choice for ectopic PitNET. However, given its special location, ectopic PitNET is difficult to operate, and completely removing part of the lesions is a challenging task. Patients who cannot receive total resection can be assisted with radiotherapy or medication after surgery. For example, bromocriptine (a dopamine antagonist) is commonly used to treat prolactin (PRL) adenomas. Ectopic PitNET is generally benign, with no recurrence or metastasis and a good prognosis [3]. After total tumor resection, the corresponding endocrine dysfunction and compression symptoms on the surrounding structures can be gradually alleviated, and the increase in hormones seems to be irrelevant to the prognosis [15].

**Figure 5:**
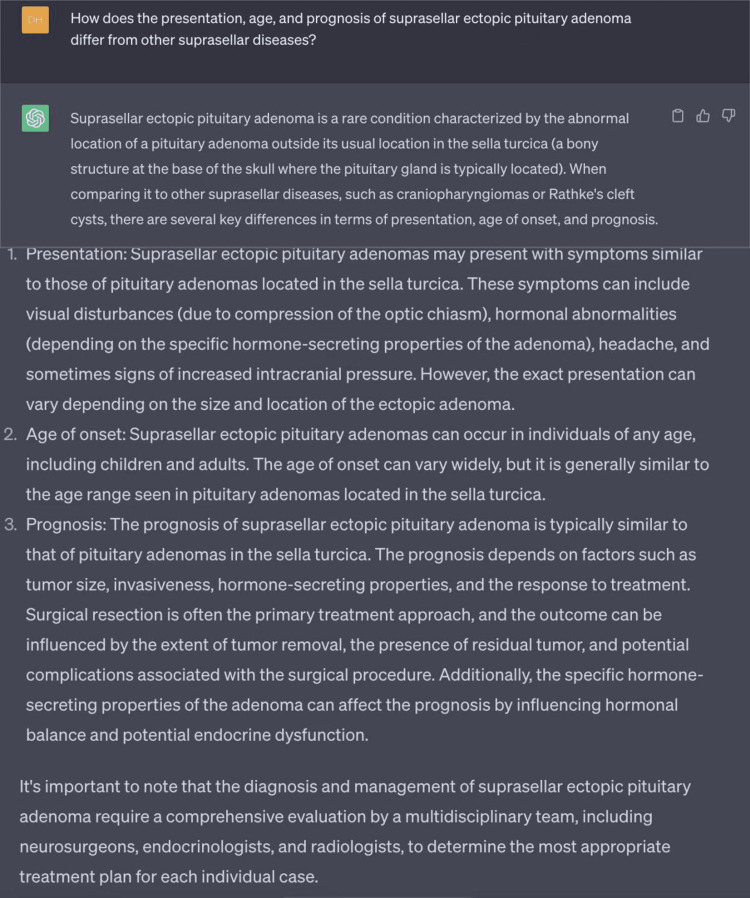
Use of ChatGPT

**Figure 6:**
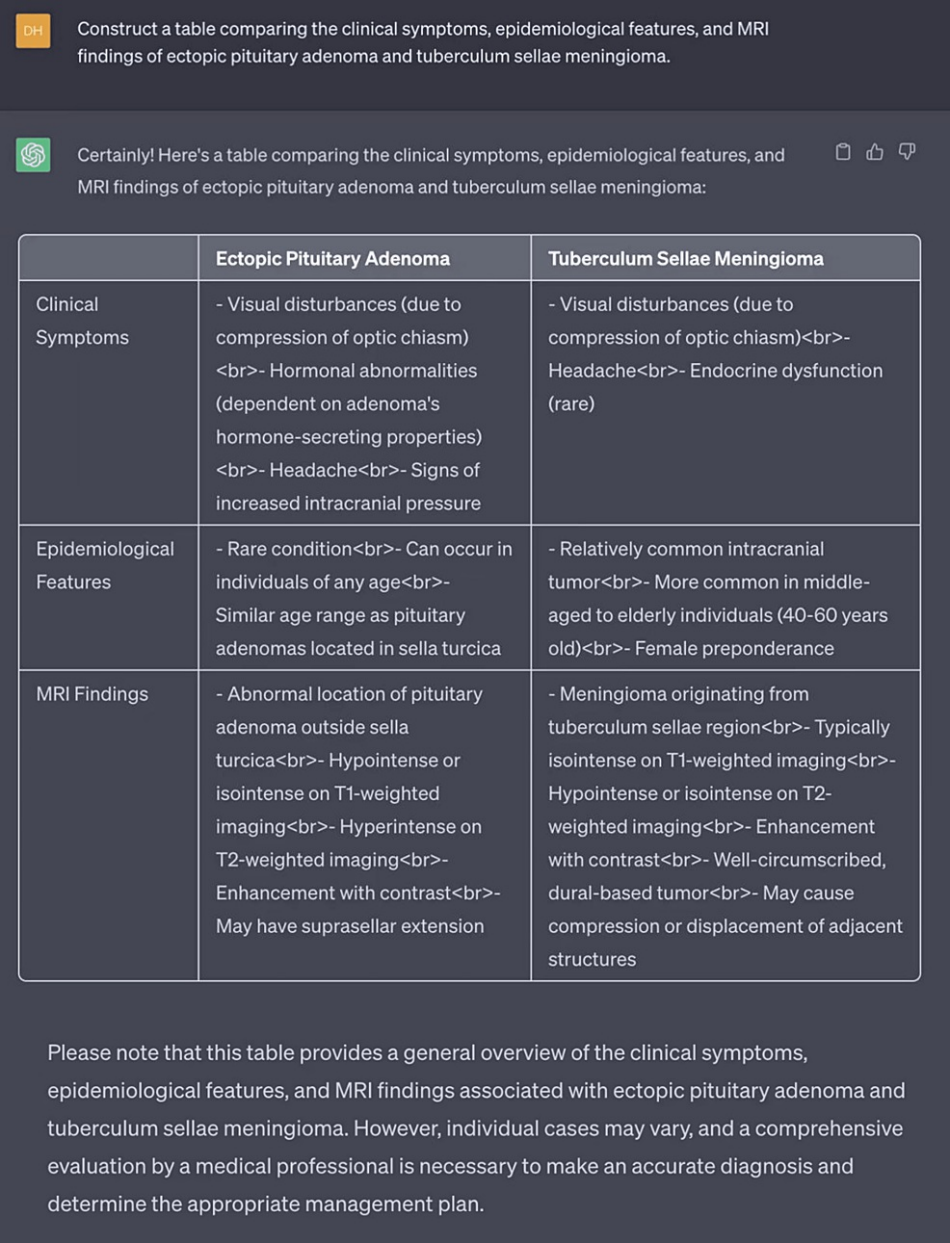
Creating a table using ChatGPT

The combination of AI and medicine is reflected in many medical fields [16]. The importance of AI in neurosurgery has significantly increased, even helping push the boundaries of neurosurgery [17]. At present, an increasing number of studies have explored the potential application of ChatGPT in various medical disciplines [18-20]. On March 14, 2023, OpenAI announced the release of a new and improved GPT-4. This new version can process images and texts, which may completely change the way humans interact with computers [21,22]. Considering the rarity of ectopic PitNETs, we extracted information based on specific questions (Table 1). ChatGTP makes reasonable relevant answers in most cases, using medical terms indexed from medical literature instead of sentences. Figure 5 and Figure 6 demonstrate the use of the ChatGPT platform.

**Table 1:** Questions posed to ChatGPT to generate this manuscript

Questions posed to ChatGPT
Definition of ectopic pituitary neuroendocrine tumor?
Embryohistological origin of ectopic pituitary neuroendocrine tumors?
Epidemiological characteristics of ectopic pituitary neuroendocrine tumors?
How to differentiate the diagnosis of a 53-year-old woman presenting with facial changes and hypertrophy of the hands and feet, MRI shows a space-occupying lesion in the suprasellar region
In the previous reports, what are the incidences of ectopic pituitary neuroendocrine tumors in different tumor sites?
What are the key points in the differential diagnosis of suprasellar ectopic pituitary neuroendocrine tumors from other suprasellar diseases?
Surgical management and prognosis of neuroendocrine tumors of the allogeneic pituitary gland?

ChatGPT is a technology for automatically generating text based on known text, which has high accuracy and the advantage of automation [23]. The generation of ChatGPT has demonstrated the effectiveness of using curated scientific and biomedical corpora for both discriminative and generative language modeling [24]. In the comparison between ectopic PitNET and other tumors in the sellar region, ChatGPT provides a basically correct answer, confirming that the answer it provides is basically consistent with the answer provided by a real doctor [1]. But usually, the answers provided by artificial intelligence do not provide any comments on the emotional and moral aspects of the case [25]. ChatGPT often produces only simple answers and repeats known facts because of the lack of reference, resulting in unverified results when specific questions that require a number or a value are asked [26]. This limitation could reduce the quality and transparency of research and fundamentally change our autonomy as human researchers. Moreover, the result is a summary of existing articles, and no clear selection method or reference source is provided, which is a defect in writing scientific articles. ChatGPT can be used to write an introductory paragraph or a specific chapter of a scientific paper, which requires relatively few references. However, when posing questions that require an in-depth understanding of the literature, ChatGPT often produces false and misleading texts [27]. Consequently, more rigorous methods must be adopted to verify the accuracy of information. These errors may be due to the lack of relevant articles in the training set of ChatGPT, the failure to extract relevant information, or the inability to distinguish between trusted and untrusted sources [28].

## Conclusions

ChatGPT concluded that suprasellar ectopic PitNETs are a rare subtype of ectopic PitNET involving the suprasellar region. The clinical presentation, diagnosis, and treatment of suprasellar ectopic PitNETs are similar to those of ectopic PitNET located elsewhere in the brain, but the procedure is more complex and challenging. The development potential of ChatGPT in medical scientific research writing is considerable, which can provide faster and more accurate support for medical scientific research writing. However, the generated content must be reviewed and verified by experts.

## Appendices

Normal range of female growth hormone (CLIA): ≤8 ng/ml

Normal reference value of insulin-like growth factor-1 in women aged 50 to 60 (CLIA): 81-238 ng/ml

## References

[1] Zhou Z (2023). Evaluation of ChatGPT’s capabilities in medical report generation. Cureus.

[2] Kung TH, Cheatham M, Medenilla A (2023). Performance of ChatGPT on USMLE: potential for AI-assisted medical education using large language models. PLOS Digit Health.

[3] Zhu Y, Liu F, Song M (2016). Extracranial ectopic pituitary adenoma: clinicopathologic analyses of three cases [Article in Chinese]. J Diag Pathol.

[4] Lu X, Hang W, Liu G (2022). Progress on diagnosis and treatment of ectopic pituitary adenoma [Article in Chinese]. Zhonghua Er Bi Yan Hou Tou Jing Wai Ke Za Zhi.

[5] Chen C, Mao W, Hou B (2015). MRI findings of ectopic pituitary adenomas [Article in Chinese]. Chin Neurosurg J.

[6] Zhu J, Wang Z, Zhang Y (2020). Ectopic pituitary adenomas: clinical features, diagnostic challenges and management. Pituitary.

[7] Johnston PC, Kennedy L, Weil RJ, Hamrahian AH (2014). Ectopic ACTH-secreting pituitary adenomas within the sphenoid sinus. Endocrine.

[8] Pasquini E, Faustini-Fustini M, Sciarretta V, Saggese D, Roncaroli F, Serra D, Frank G (2003). Ectopic TSH-secreting pituitary adenoma of the vomerosphenoidal junction. Eur J Endocrinol.

[9] Abushamat LA, Kerr JM, Lopes MB, Kleinschmidt-DeMasters BK (2019). Very unusual sellar/suprasellar region masses: a review. J Neuropathol Exp Neurol.

[10] Liang J, Libien J, Kunam V, Shao C, Rao C (2014). Ectopic pituitary adenoma associated with an empty sella presenting with hearing loss: case report with literature review. Clin Neuropathol.

[11] Liang H, Shen J, Zhao J (2016). Magnetic resonance imaging diagnosis of tuberculum sellae meningioma: a report of 59 cases. J Neurol Neurorehabil.

[12] Mu L, Zhang H, Chen Q (2016). Clinical analysis of one patient with ectopic prolactinoma in clivus and review of literature [Article in Chinese]. J Clin Neurosurg.

[13] Yang BT, Chong VF, Wang ZC, Xian JF, Chen QH (2010). Sphenoid sinus ectopic pituitary adenomas: CT and MRI findings. Br J Radiol.

[14] Das CJ, Seith A, Gamanagatti S, Goswami R (2006). On the AJR viewbox. Ectopic pituitary adenoma with an empty sella. AJR Am J Roentgenol.

[15] Thompson LD, Seethala RR, Müller S (2012). Ectopic sphenoid sinus pituitary adenoma (ESSPA) with normal anterior pituitary gland: a clinicopathologic and immunophenotypic study of 32 cases with a comprehensive review of the English literature. Head Neck Pathol.

[16] Hosny A, Aerts HJ (2019). Artificial intelligence for global health. Science.

[17] Mofatteh M (2021). Neurosurgery and artificial intelligence. AIMS Neurosci.

[18] (2023). Will ChatGPT transform healthcare?. Nat Med.

[19] Sng GG, Tung JY, Lim DY, Bee YM (2023). Potential and pitfalls of ChatGPT and natural-language artificial intelligence models for diabetes education. Diabetes Care.

[20] Grünebaum A, Chervenak J, Pollet SL, Katz A, Chervenak FA (2023). The exciting potential for ChatGPT in obstetrics and gynecology. Am J Obstet Gynecol.

[21] Lee P, Bubeck S, Petro J (2023). Benefits, limits, and risks of GPT-4 as an AI chatbot for medicine. N Engl J Med.

[22] Sanderson K (2023). GPT-4 is here: what scientists think. Nature.

[23] Ray PP (2023). ChatGPT: a comprehensive review on background, applications, key challenges, bias, ethics, limitations and future scope. Internet of Things and Cyber-Physical Systems.

[24] Luo R, Sun L, Xia Y, Qin T, Zhang S, Poon H, Liu TY (2022). BioGPT: generative pre-trained transformer for biomedical text generation and mining. Brief Bioinform.

[25] Nachshon A, Batzofin B, Beil M, van Heerden PV (2023). When palliative care may be the only option in the management of severe burns: a case report written with the help of ChatGPT. Cureus.

[26] Le DP, Hall SC (2023). Medical literature writing with ChatGPT: a rare case of choriocarcinoma syndrome with hemorrhagic brain metastases due to burned out metastatic mixed testicular cancer. Cureus.

[27] Manohar N, Prasad SS (2023). Use of ChatGPT in academic publishing: a rare case of seronegative systemic lupus erythematosus in a patient with HIV infection. Cureus.

[28] van Dis EA, Bollen J, Zuidema W, van Rooij R, Bockting CL (2023). ChatGPT: five priorities for research. Nature.

